# Liver X Receptor Agonist TO901317 Attenuates Paraquat-Induced Acute Lung Injury through Inhibition of NF-*κ*B and JNK/p38 MAPK Signal Pathways

**DOI:** 10.1155/2017/4652695

**Published:** 2017-04-05

**Authors:** Xiao Hu, Haitao Shen, Yu Wang, Min Zhao

**Affiliations:** Department of Emergency Medicine, Shengjing Hospital of China Medical University, Shenyang 110004, China

## Abstract

Paraquat (PQ) is a widely used herbicide with extremely high poisoning mortality mostly from acute lung injury (ALI) or progressive pulmonary fibrosis. Toxicity mechanisms remain unclear, but a redox cycling process that generates reactive oxygen species (ROS) is involved, as are inflammation and cell apoptosis. We established an ALI mouse model by intraperitoneal injection of PQ (28 mg/kg) and then investigated the effects of a potent liver X receptor (LXR) agonist, TO901317 (5 or 20 mg/kg), injected intraperitoneally 30 min after PQ administration. Poisoned mice exhibited severe lung tissue lesions and edema, significant neutrophilic (PMNs) infiltration, and release of the proinflammatory cytokines tumor necrosis factor-*α* (TNF-*α*) and interleukin-1*β* (IL-1*β*). PQ administration also decreased activity of antioxidases, including superoxide dismutase (SOD), catalase (CAT), and glutathione S-transferases (GSTs), and increased lipid peroxidation as evaluated by malondialdehyde (MDA) levels. PQ exposure induced upregulation of the proapoptotic gene Bax and downregulation of the antiapoptotic gene Bcl-2, leading to marked cell apoptosis in the lung tissues. TO901317 treatment reversed all these effects through inhibition of PQ-induced nuclear factor kappa B (NF-*κ*B) and JNK/p38 mitogen-activated protein kinase (MAPK) activation. The LXR agonist TO901317 had potent antioxidant, anti-inflammatory, and antiapoptotic effects against PQ-induced ALI.

## 1. Introduction

Paraquat (PQ) is a widely used herbicide with extremely high poisoning mortality due to high toxicity and lack of effective therapies. Because it is not expensive and is easy to access, the prevalence of PQ poisoning has increased dramatically: numerous fatalities have occurred in the past two decades in China [1]. Most of these patients died of acute lung injury (ALI) or progressive pulmonary fibrosis, because PQ tends to accumulate in lung tissue through the polyamine uptake system and so the pulmonary concentration becomes much higher than that in plasma and other organs [2]. The exact mechanism of PQ toxicity remains unclear, but it is well recognized that PQ exerts toxic effects through induction of a redox cycling process that results in the generation of reactive oxygen species (ROS) [2]. ROS can extract hydrogen atoms from polyunsaturated fatty acids, thus causing lipid peroxidation. This process results in structural alterations and dysfunction of cell membranes, leading to cell damage or apoptosis [3].

In addition to direct damage to pneumocytes by lipid peroxidation, ROS can also induce indirect damage though activation of several transcription factors and initiation of corresponding downstream biological processes. Nuclear factor kappa B (NF-*κ*B) is considered a master regulator of inflammation, having five components: NF-*κ*B1 (p50 and its precursor p105), NF-*κ*B2 (p52 and its precursor p100), RelA (p65), RelB, and c-Rel. Generally, NF-*κ*B is inactivated in the cytoplasm by inhibitory proteins of the I*κ*B family, including I*κ*B*α*, I*κ*B*β*, and I*κ*B*ε* [4]. ROS are typical activators of NF-*κ*B, which can induce degradation of I*κ*B*α* followed by NF-*κ*B nuclear translocation and activation of target genes, including cytokines, chemokines, and adhesion molecules [5]. Accordingly, inflammation is also an important mechanism of PQ toxicity.

After PQ exposure, both generation of ROS and activation of NF-*κ*B can induce apoptosis characterized by nuclear condensation and DNA fragmentation [6, 7]. Activation of mitogen-activated protein kinases (MAPKs) may be involved in the molecular mechanism of PQ-induced apoptosis [8]. MAPKs are protein Ser/Thr kinases that respond to various stressors and regulate cellular responses. MAPKs are composed of several distinct groups, and the most extensively studied MAPKs are extracellular signal-regulated kinases (ERK), c-Jun N-terminal kinases (JNK), and p38 kinases [9]. Both JNK and p38 can be activated by phosphorylation modification under the stimuli of various cellular stressors, such as oxidative stress and presence of TNF-*α*, and then induce proapoptotic Bax expression while inhibiting antiapoptotic Bcl-2 expression, thus resulting in activation of the Bax-mediated mitochondrial apoptosis pathway [10].

Taking into account the mechanisms of PQ toxicity mentioned above, we believe that effective treatment of PQ poisoning should be able to reverse all these effects. Liver X receptors (LXRs) are nuclear receptors (NR) that belong to the family of ligand-activated transcription factors; there are two isoforms, LXR*α* and LXR*β*. Both can be activated by natural oxysterols such as 22(R)-hydroxycholesterol, 4,25(S)-epoxycholesterol, and the synthetic agonist T0901317 [11]. LXRs were initially considered to be important regulators of cholesterol metabolism and triglyceride synthesis in various tissues [12, 13]. Recently, much evidence has identified LXRs as anti-inflammatory transcription factors [14, 15] and physiological regulators of apoptosis and phagocytosis [16–18]. In addition, it was reported that LXRs increase the activities of antioxidant enzymes, thereby reducing production of ROS and protecting against oxidative stress injury [19]. On the basis of these discoveries, we hypothesized that LXRs may be potential targets as treatment for PQ poisoning. In this study, we analyzed the role and possible molecular mechanisms of LXRs against PQ-induced ALI in mice.

## 2. Materials and Methods

### 2.1. Animals

Male wild type C57BL/6J mice (SPF grade, 8–10 weeks old, weighing 18–22 g) were purchased from the animal facility of China Medical University. All animals were housed in a comfortable environment (daily light-dark cycle, 20–25°C temperature, 40–60% humidity) and fed with rodent chow and water ad libitum. All animal experiments were conducted in compliance with China Medical University's Institutional Animal Ethics Committee and Animal Care Guidelines governing the use of experimental animals.

### 2.2. Experimental Protocols

A total of 144 mice were divided into 6 groups of 24 each and were treated as follows: (1) Control group mice were intraperitoneally injected with 0.1 mL normal saline solution twice at an interval of 30 min; (2) T0901317L group mice were intraperitoneally injected with 0.1 mL normal saline solution and T0901317 (5 mg/kg) 30 min later; (3) T0901317H group mice were intraperitoneally injected with 0.1 mL normal saline solution and T0901317 (20 mg/kg) 30 min later; (4) PQ group mice were intraperitoneally injected with PQ (28 mg/kg) and 0.1 mL normal saline solution 30 min later; (5) PQ + T0901317L group mice were intraperitoneally injected with PQ (28 mg/kg) and T0901317 (5 mg/kg) 30 min later; (6) PQ + T0901317H group mice were intraperitoneally injected with PQ (28 mg/kg) and T0901317 (20 mg/kg) 30 min later.

T0901317 was dissolved in dimethyl sulphoxide to 100 mM and diluted with normal saline. The doses of T0901317 and PQ were based on the results of previous in vivo studies [20, 21]. At 6, 12, 24, and 72 h after PQ administration, 6 mice in each group were sacrificed for testing.

### 2.3. Lung Wet-to-Dry (W/D) Weight Ratios

Mice were sacrificed 72 h after PQ exposure and right lower lobes were excised. Each lung was weighed and then placed in an oven at 80°C for 48 h to obtain the “dry” weight. The ratio of the weight of the wet lung to the weight of the dry lung was calculated to assess tissue edema.

### 2.4. Histopathology

Mice were sacrificed 72 h after PQ administration. The lung tissues were fixed in 4% paraformaldehyde for 48 h at 4°C, dehydrated through a graded ethanol series, and embedded in paraffin. After dewaxing, samples were sectioned and stained with hematoxylin and eosin according to established methods and then evaluated by light microscopy using a semiquantitative scoring method. Lung injury was graded in a blinded fashion from 0 (normal) to 4 (severe) for interstitial inflammation, neutrophil infiltration, congestion, and edema. The total lung injury score was calculated by adding up the individual scores for each category.

### 2.5. Bronchoalveolar Lavage Fluid (BALF) Collection and Cytokine Measurement

At 6, 12, 24, and 72 h after PQ administration, 6 mice in each group were euthanized and BALF was collected by washing 3 times with 1 mL phosphate buffered saline (PBS) solution after cannulation of the left trachea. The collected BALF was centrifuged at 1000*g* for 10 min. The cell-free supernatant fluid was collected for measurement of cytokines, including tumor necrosis factor-*α* (TNF-*α*) and interleukin-1*β* (IL-1*β*), using enzyme-linked immunosorbent assay (ELISA) kits (Wuhan Boster Bio-Engineering Co., Ltd., Wuhan, China), according to the manufacturers' instructions.

### 2.6. Measurement of Myeloperoxidase (MPO) Activity

The activity of MPO in lung tissue was determined by using an MPO kit (Nanjing Jiancheng Bioengineering Institute, Nanjing, China) at 6, 12, 24, and 72 h after PQ exposure. MPO activity was determined by measuring the H_2_O_2_-dependent oxidation of 3,3′-dimethoxybenzidine and was expressed as units/g of protein.

### 2.7. Measurement of Superoxide Dismutase (SOD), Catalase (CAT), and Glutathione S-Transferase (GST) Activities

At 6, 12, 24, and 72 h after PQ exposure, lung tissues were homogenized in PBS (pH 7.4) to obtain a 10% (w/v) homogenate. Homogenates were centrifuged at 12000 ×g for 30 min at 4°C. Supernatants were assayed for enzymatic activity using kits (Nanjing Jiancheng Bioengineering Institute, Nanjing, China) according to the manufacturer's instructions.

### 2.8. Malondialdehyde (MDA) Measurement

At 6, 12, 24, and 72 h after PQ exposure, lipid peroxidation was evaluated by determining the MDA level, using a kit for measurement by the thiobarbituric acid (TBA) method (Nanjing Jiancheng Bioengineering Institute, China), according to the manufacturer's instructions.

### 2.9. Western Blot Analysis

At 72 h after PQ exposure, lung tissues were harvested and homogenized and then processed using a Nuclear and Cytosolic Protein Extraction Kit (Beyotime Institute of Biotechnology) according to the manufacturer's instructions. Protein concentrations were determined using a BCA protein assay kit (Beyotime Institute of Biotechnology) and samples containing equal amounts of protein were subjected to a 12% sodium dodecyl sulphate polyacrylamide gel (SDS-PAGE). Subsequently, proteins were transferred onto polyvinylidene difluoride membranes and incubated at 4°C overnight with primary antibodies (all from Abcam, Cambridge, UK, except as noted) specific for ABCA1 (1 : 500), NF-*κ*Bp65 (1 : 400), I*κ*B-*α* (1 : 1000), phosphor-p38 (1 : 1000 CST, Danvers, MA, USA), phosphor-JNK (1 : 1000), Bax (1 : 2000), and Bcl-2 (1 : 400). After washing, the membrane was incubated with the secondary antibody at room temperature for 1 h. *β*-Actin or histone H3 was used as the loading control. Signals were detected with an enhanced chemiluminescence system (Amersham Pharmacia, NJ, USA) and quantitative analysis was performed using Gel-Pro-Analyzer 6.3 software.

### 2.10. Immunohistochemistry

Mice were killed 72 h after PQ administration and lung tissues were processed for sectioning as described above. Paraffin-embedded sections were dewaxed, hydrated, and incubated with 3% H_2_O_2_ in methanol to block endogenous peroxidase. After incubation with 2% (v/v) normal goat serum in PBS for 20 min, the sections were incubated with primary antibodies specific for NF-*κ*B p65 (1 : 200) and phosphor-JNK (1 : 100) (Abcam) and phosphor-p38 (1 : 200) (CST) overnight at 4°C. The sections were then washed with PBS and incubated with secondary antibody at room temperature for 30 min. Reaction products were labeled by a horseradish peroxidase-conjugated antibody, visualized with diaminobenzidine (DAB), and counterstained with hematoxylin.

### 2.11. Terminal Deoxynucleotidyl Transferase-Mediated UTP End Labeling (TUNEL) Assay

TUNEL assays were conducted by using a TUNEL assay kit (In Situ Cell Death Detection Kit, POD, Roche, Switzerland) according to the manufacturer's instructions. Briefly, after pretreatment with proteinase K, paraffin-embedded sections were dewaxed, hydrated, and incubated with 3% H_2_O_2_ in methanol to block endogenous peroxidase. Next, sections were incubated at 37°C for 1 h with a reaction mixture containing terminal deoxynucleotidyl transferase and biotinylated deoxyuridine triphosphate (dUTP). After washing with PBS, the sections were incubated with anti-horseradish peroxidase-conjugated antibody, visualized with diaminobenzidine, and counterstained with hematoxylin.

### 2.12. Statistical Analyses

The data are expressed as mean ± SD. Statistical analyses were carried out using SPSS 17.0. One-way ANOVA followed by least significance difference (LSD) determination was used to compare results of the different treatment groups. Differences having *p* values < 0.05 were considered statistically significant.

## 3. Results

### 3.1. Mortality and Changes of Body Weight

No death was observed in the duration of this experiment. However, diarrhea, anorexia, adipsia, and dyspnea were present in the mice of PQ group resulting in significant weight loss.

As shown in Table 1, there was no significant difference in body weight among six groups at beginning of the experiment. At 72 h after PQ exposure, the body weight of mice in PQ group significantly declined compared with control group. TO901317 treatment relieved PQ-induced symptoms and weight loss in a dose dependent manner.

### 3.2. Lung W/D Weight Ratios

**Figure 1:**
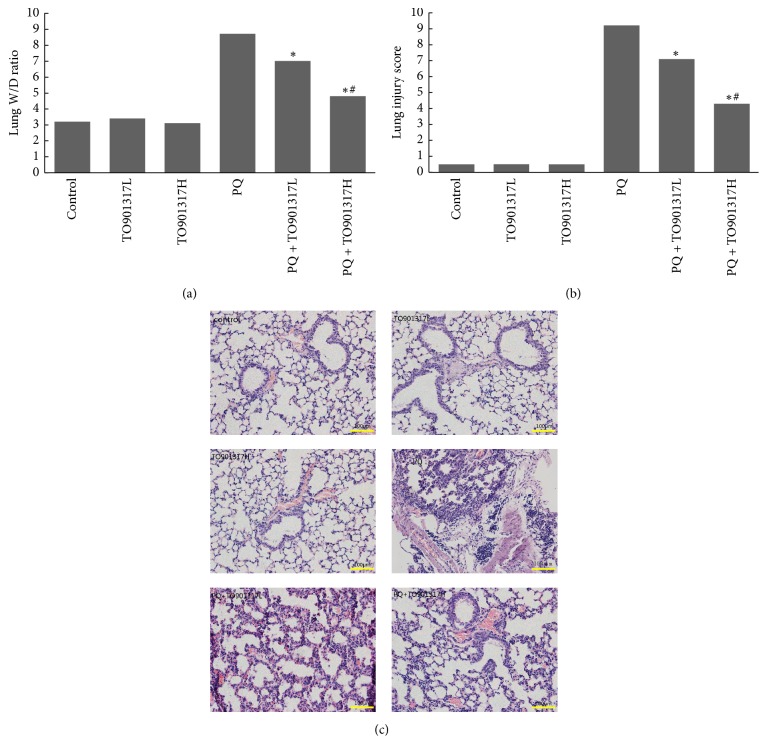
Effects of TO901317 on lung W/D ratio and histopathological changes in lung tissues. The lung W/D ratio (a) and lung histological evaluation (b, c, HE staining, 200x) were determined 72 h after PQ administration. TO901317L: TO901317 at the low dose of 5 mg/kg; TO901317H: TO901317 at the high dose of 20 mg/kg. The values presented are the mean ± SD (*n* = 6). ^**∗**^*p* < 0.05 versus PQ group. ^#^*p* < 0.05 versus PQ + TO901317L group.

The lung W/D weight ratios were assessed to evaluate the degree of pulmonary edema at 72 h after PQ exposure. As shown in Figure 1(a), the ratios in the PQ group were significantly higher than those in the other groups. TO901317 treatment attenuated the rise of lung W/D weight ratios in a dose dependent manner.

### 3.3. Histopathological Changes in the Lung Tissues

Histopathological changes were determined by HE staining of lung tissues harvested 72 h after PQ exposure. As shown in Figures 1(b) and 1(c), the control and TO901317L and TO901317H groups exhibited normal pulmonary structure without obvious differences. PQ exposure induced significant histological lesions, including alveolar hemorrhage, alveolar wall thickening, interstitial edema, cellular infiltration, and even structural collapse, compared to the control group. However, T0901317 treatment significantly attenuated those PQ-induced histological lesions in a dose dependent manner.

### 3.4. Expression of ABCA1 in Lung Tissues

**Figure 2:**
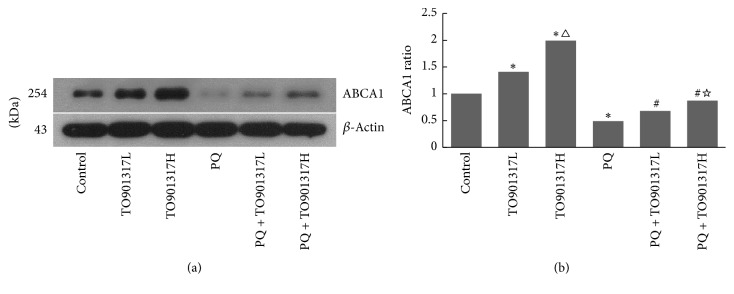
The expression of ABCA1 in lung tissues. We collected the lung tissues to perform Western blot to evaluate the expression of ABCA1 at 72 h after PQ administration. TO901317L: TO901317 at the low dose of 5 mg/kg; TO901317H: TO901317 at the high dose of 20 mg/kg. The values presented are the mean ± SD (*n* = 6). ^**∗**^*p* < 0.05 versus control group, ^△^*p* < 0.05 versus TO901317L group, ^#^*p* < 0.05 versus PQ group, and ^☆^*p* < 0.05 versus PQ + TO901317L group.

To evaluate the activation of LXRs, we determined the expression of LXRs target gene ABCA1 by Western blot analysis. As shown in Figures 2(a) and 2(b), TO901317 significantly increased the expression of ABCA1 regardless of the PQ treatment in a dose dependent manner. This result indicates that the LXRs were effectively activated in lung tissues of mice.

### 3.5. Inflammatory Cytokine Release

**Figure 3:**
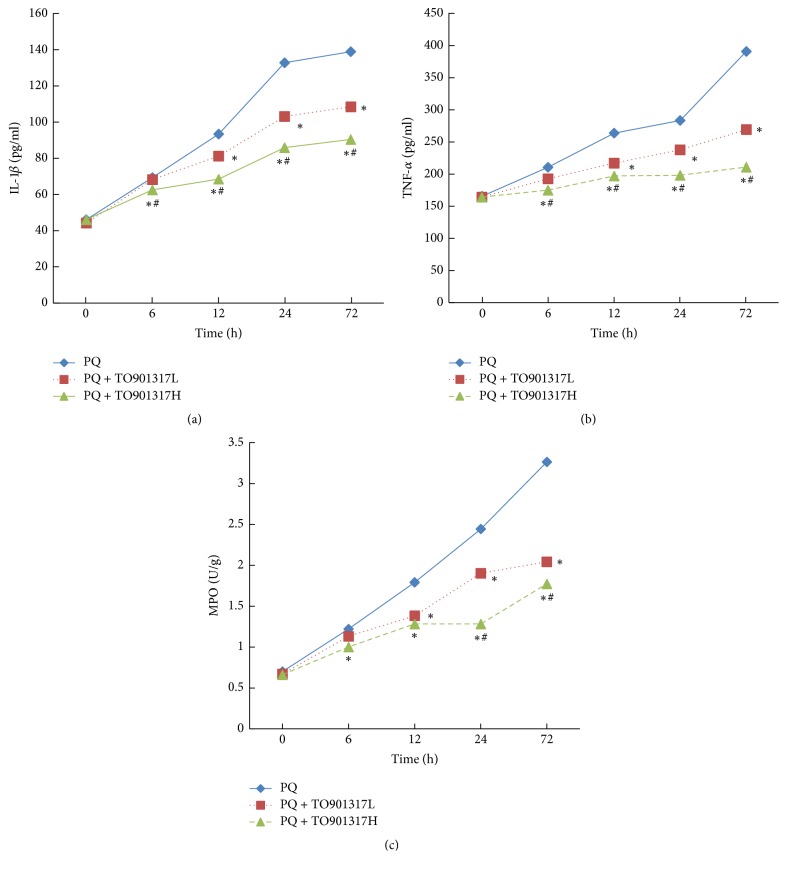
Effects of TO901317 on inflammatory cytokine release and MPO activity. BALF and lung tissues were collected after PQ administration for 6, 12, 24, and 72 h to analyze IL-1*β* (a), TNF-*α* (b), and MPO activity (c). TO901317L: TO901317 at the low dose of 5 mg/kg; TO901317H: TO901317 at the high dose of 20 mg/kg. The values presented are the mean ± SD (*n* = 6). ^**∗**^*p* < 0.05 versus PQ group. ^#^*p* < 0.05 versus PQ + TO901317L group.

ELISAs were performed to analyze the levels of TNF-*α* and IL-1*β* in the lung tissues at 6, 12, 24, and 72 h after PQ administration. As shown in Figures 3(a) and 3(b), IL-1*β* and TNF-*α* levels in the PQ group were dramatically increased compared to those in the control group. The increases of TNF-*α* and IL-1*β* were markedly attenuated, in a dose dependent manner, by T0901317 treatment.

### 3.6. MPO Activity

As shown in Figure 3(c), PQ administration resulted in a dramatic increase in MPO activity, an indicator of the degree of infiltration of PMNs [22]. However, compared with the PQ group, T0901317 treatment significantly reduced MPO activity, in a dose dependent manner.

### 3.7. Lipid Peroxidation and Antioxidant Enzyme Activities

**Figure 4:**
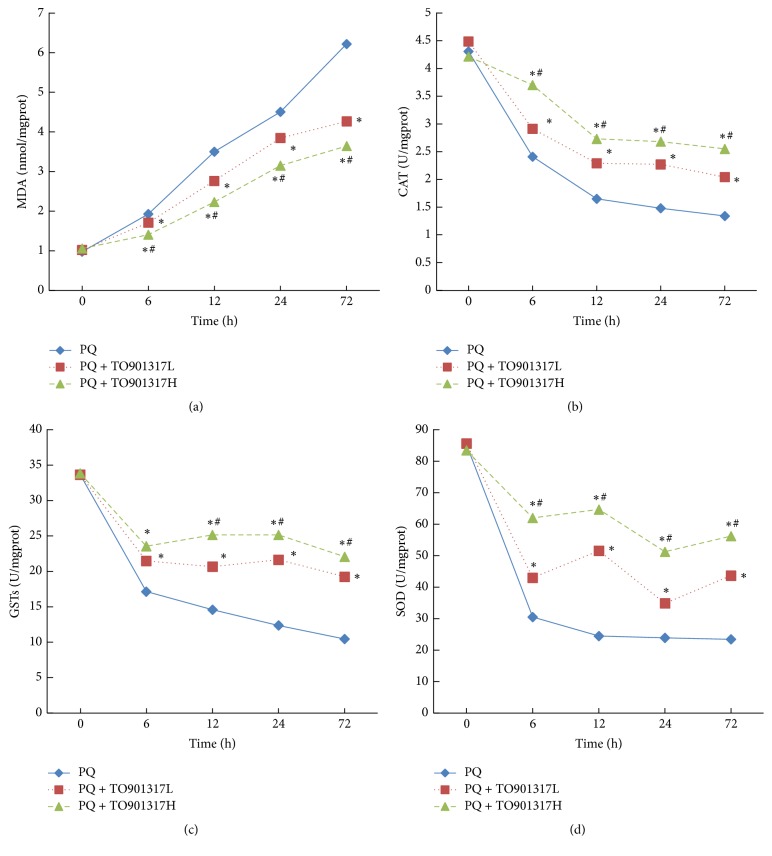
Effects of T0901317 on lipid peroxidation and antioxidant enzymes activities. The lung tissues were collected and homogenized to measure MDA (a) level and the activities of CAT (b), GSTs (c), and SOD (d) at 6, 12, 24, and 72 h after PQ administration. TO901317L: TO901317 at the low dose of 5 mg/kg; TO901317H: TO901317 at the high dose of 20 mg/kg. The values presented are the mean ± SD (*n* = 6). ^**∗**^*p* < 0.05 versus PQ group. ^#^*p* < 0.05 versus PQ + TO901317L group.

We measured MDA levels and antioxidant enzyme activities to evaluate the antioxidant capacity of lung tissues. As shown in Figure 4(a), the MDA level, an indicator of lipid peroxidation, was significantly increased in the PQ group. Treatment with T0901317 attenuated the PQ-induced increase in MDA levels, in a dose dependent manner.

Alterations of antioxidant capacity were evaluated by antioxidant enzyme activities in lung tissues. Compared with the control group, PQ administration resulted in a significant decrease in antioxidant enzyme activities, including those of SOD, CAT, and GSTs. However, T0901317 treatment counteracted these effects, in a dose dependent manner (Figures 4(b), 4(c), and 4(d)).

### 3.8. NF-*κ*B Activation and I*κ*B-*α* Expression

**Figure 5:**
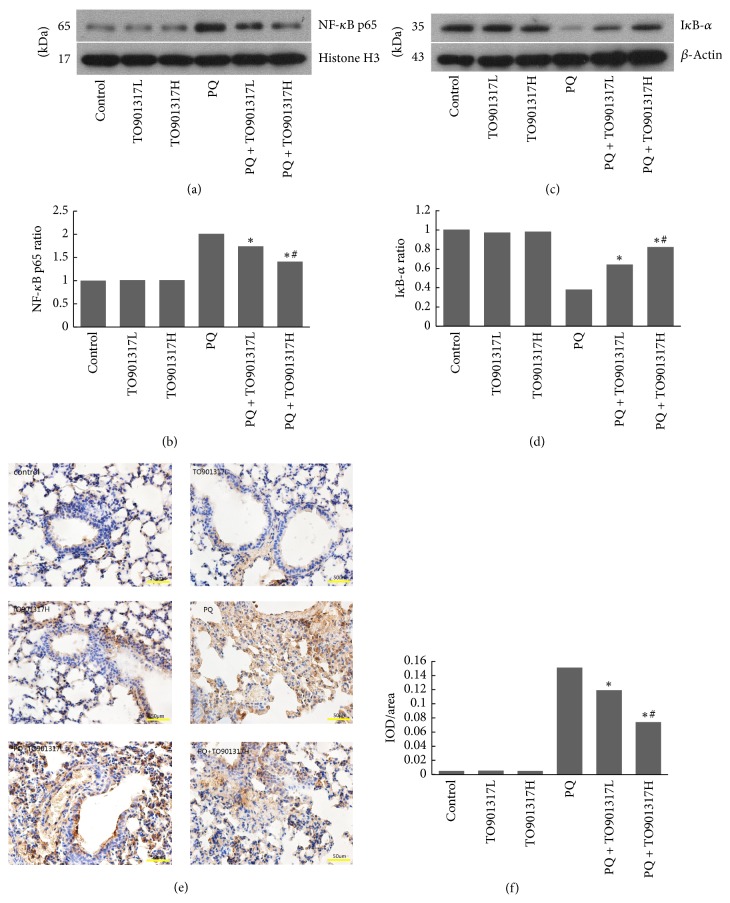
Effect of T0901317 on NF-*κ*B activation and I*κ*B-*α* degradation in lung tissues of mice. Lung tissues were collected to determine the nuclear expression of NF-*κ*B p65 by Western blot (a, b) and immunohistochemistry (e, f ×400) 72 h after PQ administration. We also detected the expression of I*κ*B-*α* by Western blot (c, d) after PQ administration for 72 h. TO901317L: TO901317 at the dose of 5 mg/kg; TO901317H: TO901317 at the dose of 20 mg/kg. The values presented are the mean ± SD (*n* = 5). ^**∗**^*p* < 0.05 versus PQ group. ^#^*p* < 0.05 versus PQ + TO901317L group.

We evaluated NF-*κ*Bp65 and I*κ*B-*α* expression to investigate the possible cellular mechanisms whereby T0901317 treatment attenuates PQ-induced ALI. As shown in Figures 5(a)–5(f), PQ exposure induced a significant increase of NF-*κ*B p65 expression in nuclear extracts and a marked decrease of I*κ*B-*α* expression in cytoplasm, in comparison with the control group. This phenomenon indicated that PQ induced degradation of I*κ*B-*α*, and thus NF-*κ*B p65 was released and transferred to the nucleus, resulting in activation of the downstream proinflammatory gene. However, this effect was significantly inhibited by treatment with T0901317, in a dose dependent manner.

### 3.9. JNK and p38 MAPK Expression

**Figure 6:**
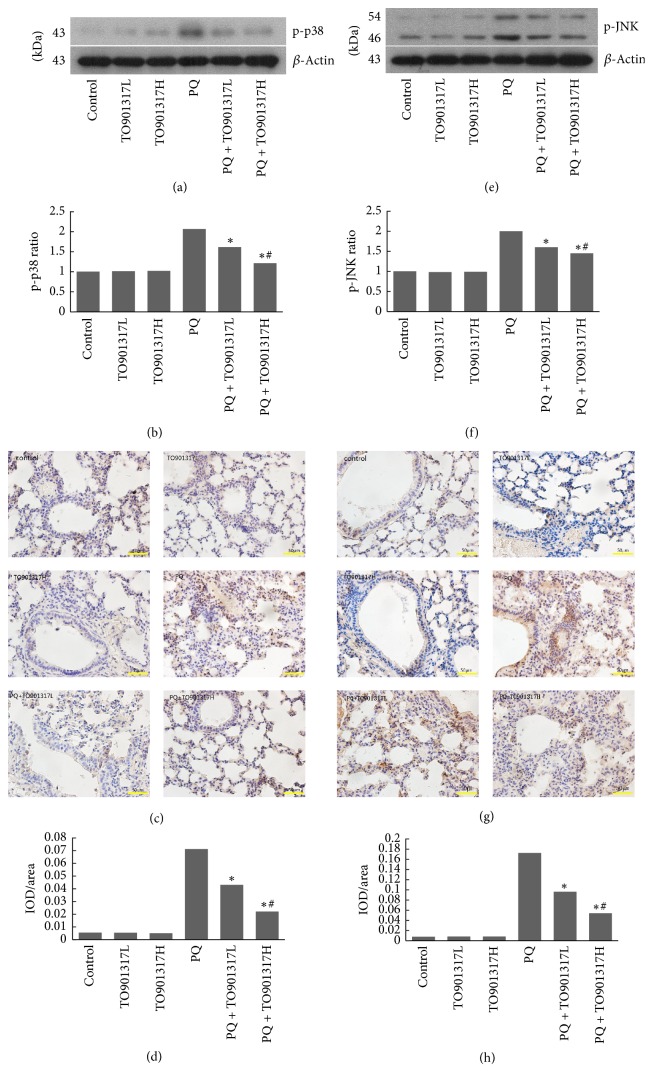
Effect of T0901317 on JNK/p38 MAPKs signal pathway in lung tissues of mice. Lung tissues were collected after PQ administration for 72 h to determine the expression of p-p38 (a, b, c, d) and p-JNK (e, f, g, h) by Western blot and immunohistochemistry. TO901317L: TO901317 at the dose of 5 mg/kg; TO901317H: TO901317 at the dose of 20 mg/kg. The values presented are the mean ± SD (*n* = 5). ^**∗**^*p* < 0.05 versus PQ group; ^#^*p* < 0.05 versus PQ + TO901317L group.

The activation of JNK and p38 MAPK was assessed to further investigate the cellular mechanisms by which T0901317 treatment may attenuate PQ-induced ALI. As shown in Figures 6(a)–6(h), PQ administration caused a significant increase in phosphorylation of JNK and p38 MAPK in the lung tissues, compared with the control group. Treatment with T0901317 significantly inhibited the activation of JNK and p38 MAPK in a dose dependent manner, compared to the PQ group.

### 3.10. Apoptosis in the Lung Tissues

**Figure 7:**
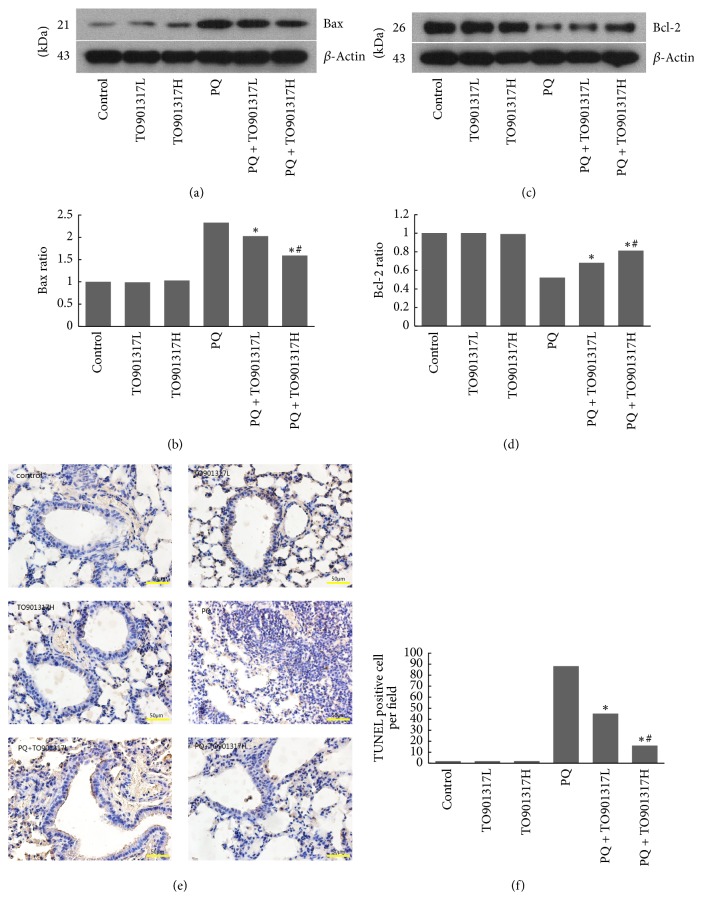
Effects of T0901317 on the expression of Bax and Bcl-2 and apoptosis. The lung tissues were collected 72 h after PQ administration to determine the expression of Bax (a, b) and Bcl-2 (c, d) by Western blot. PQ-induced apoptosis was evaluated by TUNEL-like staining (e, f) 72 h after PQ administration. TO901317L: TO901317 at the low dose of 5 mg/kg; TO901317H: TO901317 at the high dose of 20 mg/kg. The values presented are the mean ± SD (*n* = 5). ^**∗**^*p* < 0.05 versus PQ group; ^#^*p* < 0.05 versus PQ + TO901317L group.

TUNEL-like staining was performed to evaluate apoptosis in the lung tissues. As shown in Figures 7(e) and 7(f), the PQ group exhibited greatly increased positive staining, indicating apoptosis, compared with that observed in the control group. However, T0901317 treatment significantly inhibited PQ-induced apoptosis in the lung tissues.

### 3.11. Bax and Bcl-2 Expression

To estimate the apoptotic signal transduction, we assessed the expression of Bax and Bcl-2 by Western blot. As shown in Figures 7(a)–7(d), PQ administration significantly upregulated Bax expression and downregulated Bcl-2 expression in the lung tissues. However, T0901317 treatment significantly prevented PQ-induced regulation of the apoptotic signal transduction.

## 4. Discussion

It has been shown in many previous studies [23, 24] that PQ can be actively transported against a marked concentration gradient and accumulated into type I and type II alveolar epithelial cells through the polyamine uptake system. Accumulation of PQ induces significant damage and destruction to the pulmonary epithelium, followed by dysfunction of gas exchange and lack of surfactant, resulting in histopathological alterations, such as alveolitis, and clinical manifestations, including ALI and ARDS [2]. Endothelium damage also plays an important role in the pathophysiological process of PQ toxicity. Damaged pulmonary capillary endothelium may be a key factor contributing to the production of pulmonary hemorrhage, proteinaceous edema, and the infiltration of inflammatory cells into the interstitium and alveoli [25, 26]. In accordance with these observations, we found that PQ exposure induced significant alveolitis characterized by alveolar hemorrhage, alveolar wall thickening, alveolar and interstitial edema, inflammatory cell infiltration, and even structural collapse. As a consequence of increased capillary permeability resulting from PQ-induced endothelium damage, the BALF protein concentration was significantly increased in the PQ group. Protein leakage further aggravated pulmonary edema, as evidenced by the higher W/D weight ratios in the PQ group compared to those of the control group. The PQ-induced increase of capillary permeability also resulted in significant inflammatory cell infiltration accompanied by a marked increase of the proinflammatory cytokines TNF-*α* and IL-1*β*. It is noteworthy that T0901317 treatment attenuated all these biochemical and histopathological parameters of PQ toxicity in a dose dependent manner, which suggests that LXR activation may be a potential target as a new treatment of PQ poisoning. However, what is the mechanism by which T0901317 confers potent protection against PQ-induced ALI?

As mentioned above, ROS generation and lipid peroxidation play an important role in the pathogenesis of PQ poisoning. LXR activation has been reported to effectively inhibit the generation of ROS and lipid peroxidation under various stress conditions [27, 28]. In this study, we measured MDA levels to evaluate the magnitude of PQ-induced lipid peroxidation. PQ exposure caused an obvious rise in MDA levels, while T0901317 treatment significantly alleviated this rise. This effect was also confirmed indirectly by the amelioration of antioxidant enzyme activities. The PQ-induced decline in the activities of antioxidant enzymes, including SOD, CAT, and GSTs, was significantly attenuated by T0901317 treatment, in a dose dependent manner. However, the precise mechanism by which LXRs inhibit PQ-induced generation of ROS remains unclear, and future studies are necessary. Furthermore, we cannot exclude the possibility that LXR-mediated remission of inflammation was indirectly associated with the regulation of oxidative stress.

According to the previous literature, LXR-mediated remission of inflammation is mainly due to the suppression of NF-*κ*B activation [29, 30]. A similar result was observed in our work. T0901317 treatment reversed PQ-induced degradation of I*κ*B-*α* and nuclear translocation of NF-*κ*B p65 in the lung tissues. As important target genes of NF-*κ*B, adhesion molecules and chemokines play significant roles in transendothelial migration and recruitment of PMNs into lung tissue [31, 32]. In this study, we determined the activity of MPO to estimate the degree of infiltration of PMNs. Consistent with NF-*κ*B expression, T0901317 treatment obviously inhibited the PQ-induced increase of MPO activity in the lung tissues. This effect was also evidenced by histopathological analysis. Cytokines are another set of important target genes of NF-*κ*B, as evidenced by the predominance of TNF-*α* and IL-1*β* in the early stage of inflammation [33]. The release of TNF-*α* and IL-1*β* leads to secondary inflammatory cascades that increase levels of ROS, cytokines, and adhesion molecules, resulting in further damage to lung tissue [34]. In this study, a similar pattern of the inhibitory effect of T0901317 was observed when TNF-*α* and IL-1*β* levels were measured. Considering that TNF-*α* and IL-1*β* induce the production of ROS, we infer that LXRs regulate oxidative stress at least partially through the inhibition of NF-*κ*B expression.

Along with effects on oxidant stress and inflammation, activation of LXRs also contributes to amelioration of PQ-induced apoptosis. In the present study, we performed TUNEL assay to assess cell apoptosis in lung tissues. Our results showed that TO901317 treatment significantly inhibited PQ-induced apoptosis. Regarding the apoptotic signal pathway, JNK and p38 MAPK were described as the mediator of PQ-induced apoptosis [8] mainly through promoting Bax expression while suppressing Bcl-2 expression [10]. The increased Bax/Bcl-2 ratio leads to reduction of the mitochondrial transmembrane potential and release of proapoptotic protein from the mitochondria into the cytosol, ultimately resulting in apoptosis [35]. As already observed in a previous study [36], PQ exposure caused a significant increase in the expressions of JNK and p38 MAPK, as well as subsequent upregulation of Bax and downregulation of Bcl-2. The activation of JNK and p38 MAPK may be associated with PQ-induced oxidant stress and release of cytokines [9]. TO901317 treatment significantly reversed PQ-induced activation of JNK and p38 MAPK and thus inhibited PQ-induced regulation of Bax and Bcl-2, in a dose dependent manner. This result indicates that LXRs confer a protective effect against PQ-induced apoptosis through blockade of the JNK and p38 MAPK signal pathway. Moreover, it is reported that activation of JNK and p38 MAPK also leads to generation of various cytokines, including IL-1*β*, TNF-*α*, IL-8, and IL-6 [37, 38], and upregulation of the NF-*κ*B signal pathway [39]. This suggests that the anti-inflammatory effect of LXRs may be achieved partially through inhibition of the JNK and p38 MAPK signal pathway.

Hypercholesterolemia is well known as a risk factor of oxidative stress which leads to increase of excess formation of ROS [40, 41] and diminished endogenous antioxidant capacity [42, 43]. Since Wang et al. [44] reported that the plasma levels of cholesterol significantly increased in PQ poisoned patients in comparison to healthy volunteers, we hypothesize that PQ toxicity may also be associated with the changes of cholesterol metabolism. Considerable evidence indicates that LXRs are crucial regulators of cholesterol metabolism and play an important role in the pathway of reverse cholesterol transport, in which excess cholesterol is transported in high-density lipoprotein (HDL) particles from peripheral cells to the liver for excretion in bile [45]. Accordingly, this effect may be beneficial in protecting against PQ toxicity. Although PQ mainly accumulate in the lung tissues, PQ toxicity is not limited to the lungs and involves other tissues such as brain, liver, and kidney [46–48]. Both LXR*α* and LXR*β* show high expression in these tissues. LXR*α* is expressed in liver, spleen, kidney, adipose tissue, and small intestine, whereas LXR*β* is ubiquitously expressed [11]. Based on the results of the present study, we suppose that LXR activation may also be beneficial in extrapulmonary organs. However, further studies are necessary to prove it.

In summary, our results clearly demonstrated that treatment with the LXR agonist TO901317 had a significant protective effect against PQ-induced acute lung injury. This effect may be due to antioxidative, anti-inflammatory, and antiapoptotic properties effected through inhibition of the NF-*κ*B and JNK/p38MAPK signal pathways. This study suggests that drug activation of LXRs may represent a new therapeutic strategy for treatment of PQ-induced ALI.

## Figures and Tables

**Table 1:** Changes of body weight in mice.

Time (h)	Weight (g)					
	Control	TO901317L	TO901317H	PQ	PQ + TO901317L	PQ + TO901317H
0 h	20.4 ± 0.8	20.3 ± 0.7	19.9 ± 0.7	20.1 ± 0.6	20.2 ± 0.8	20.4 ± 0.7
72 h	21.3 ± 0.9	21.5 ± 0.8	21.2 ± 0.6	15.8 ± 2.1^**∗**^	17.5 ± 1.8^#^	18.9 ± 1.7^#☆^

The values presented are the mean ± SD (*n* = 6). ^**∗**^*p* < 0.05 versus control group, ^#^*p* < 0.05 versus PQ group, and ^☆^*p* < 0.05 versus PQ + TO901317L group.
